# Postoperative pulmonary complications with adjuvant regional anesthesia versus general anesthesia alone: a sub-analysis of the Perioperative Research Network study

**DOI:** 10.1186/s12871-022-01679-5

**Published:** 2022-05-03

**Authors:** Karsten Bartels, Gyorgy Frendl, Juraj Sprung, Toby N. Weingarten, Balachundhar Subramaniam, Ricardo Martinez Ruiz, Jae-Woo Lee, William G. Henderson, Angela Moss, Alissa Sodickson, Jadelis Giquel, Marcos Francisco Vidal Melo, Ana Fernandez-Bustamante, for the Perioperative Research Network (PRN) investigators, David Amar, Karsten Bartels, James Blum, Lee-Lynn Chen, Dawn Desiderio, David Josh Douin, Ana Fernandez-Bustamante, Matthias Eikermann, Gyorgy Frendl, Jadelis Giquel, Loreta Grecu, Ravindra Alok Gupta, Suzanne Karan, Daryl J. Kor, Jae-Woo Lee, Ricardo Martinez Ruiz, Guido Musch, Juraj Sprung, Balachundhar Subramaniam, Alissa Sodickson, Pedro Tanaka, Marcos Francisco Vidal Melo, Jonathan P. Wanderer, Toby N. Weingarten

**Affiliations:** 1 Department of Anesthesiology, University of Colorado School of Medicine, 12631 E 17th Ave, AO-1 bldg, R2012, MS 8202, Aurora, CO 80045 USA; 2 University of Nebraska Medical Center, Omaha, NE USA; 3 Brigham and Women’s Hospital, Boston, MA USA; 4 Mayo Clinic, Rochester, MN USA; 5 Beth Israel Deaconess Medical Center, Boston, MA USA; 6 University of Miami, Palmetto Bay, FL USA; 7 University of California San Francisco, San Francisco, CA USA; 8 Adult and Children Outcomes Research and Delivery Systems (ACCORDS), University of Colorado School of Medicine, Aurora, CO USA; 9 Columbia University Irving Medical Center, New York, NY USA

**Keywords:** Regional anesthesia, Adjuvant regional anesthesia, General anesthesia, Surgical outcomes, Postoperative pulmonary complications

## Abstract

**Background:**

Adjuvant regional anesthesia is often selected for patients or procedures with high risk of pulmonary complications after general anesthesia. The benefit of adjuvant regional anesthesia to reduce postoperative pulmonary complications remains uncertain. In a prospective observational multicenter study, patients scheduled for non-cardiothoracic surgery with at least one postoperative pulmonary complication surprisingly received adjuvant regional anesthesia more frequently than those with no complications. We hypothesized that, after adjusting for surgical and patient complexity variables, the incidence of postoperative pulmonary complications would not be associated with adjuvant regional anesthesia.

**Methods:**

We performed a secondary analysis of a prospective observational multicenter study including 1202 American Society of Anesthesiologists physical status 3 patients undergoing non-cardiothoracic surgery. Patients were classified as receiving either adjuvant regional anesthesia or general anesthesia alone. Predefined pulmonary complications within the first seven postoperative days were prospectively identified. Groups were compared using bivariable and multivariable hierarchical logistic regression analyses for the outcome of at least one postoperative pulmonary complication.

**Results:**

Adjuvant regional anesthesia was performed in 266 (22.1%) patients and not performed in 936 (77.9%). The incidence of postoperative pulmonary complications was greater in patients receiving adjuvant regional anesthesia (42.1%) than in patients without it (30.9%) (site adjusted *p* = 0.007), but this association was not confirmed after adjusting for covariates (adjusted OR 1.37; 95% CI, 0.83–2.25; *p* = 0.165).

**Conclusion:**

After adjusting for surgical and patient complexity, adjuvant regional anesthesia versus general anesthesia alone was not associated with a greater incidence of postoperative pulmonary complications in this multicenter cohort of non-cardiothoracic surgery patients.

## Background

Postoperative pulmonary complications (PPCs) are a leading cause of surgical morbidity and increased healthcare costs [1]. The Perioperative Research Network (PRN) investigators recently reported the incidence and impact of PPCs among 1202 ASA physical status 3 patients undergoing non-cardiothoracic surgery [2]. Patients requiring at least 2 h of general anesthesia (GA) with mechanical ventilation were eligible for this multicenter prospective observational study [2]. In the univariate comparison, patients who had ≥1 PPC were more likely to have received adjuvant regional anesthesia (combined regional + general anesthesia, RA + GA) than patients with no PPCs [2, 3]. This was surprising, in light of the presumed benefits of RA to preserve an adequate postoperative respiratory function [4–6]. Because RA was not a controlled intervention in the original study, the observed association between RA and the incidence of PPCs warrants further analysis for any confounding factors influencing such statistical finding.

The landmark Australian MASTER trial in 915 high-risk patients undergoing major abdominal surgery found a significantly lower incidence of respiratory failure in patients receiving perioperative epidural analgesia compared to those with a systemic opioid-based regimen (23% vs. 30%, respectively) [7]. Another high-quality prospective randomized controlled trial testing the effect of epidurally administered opioids compared to systemically administered opioids found no significant reduction in death and major complications 30 days postoperatively amongst 1021 patients undergoing major abdominal surgery, although the duration of mechanical ventilation was shorter in the epidural analgesia group [8]. A meta-analysis of 141 trials and 9559 patients found that neuraxial blockade reduced respiratory depression by 59% and pneumonia by 39% (both *P* < 0.001) [4]. For continuous peripheral nerve blocks, a reduction in opioid-associated side effects as well as superior postoperative analgesia is well established [9]. Although upper extremity nerve blocks can lead to impaired respiratory muscle function, e.g., through transient blockade of the phrenic nerve [10], reduced requirements of perioperative opioids by patients receiving RA is thought to counterbalance such adverse effects [11].

Whether the combination of RA + GA as opposed to only GA is a marker of surgical complexity and/or increased patient comorbidities or possibly associated with other adverse exposures is unknown [3]. Here, we performed a secondary analysis of the PRN PPC study dataset to explore the association between the incidence of PPCs and the presence of adjuvant RA [2]. We tested the hypothesis that the incidence of ≥1 PPC was not associated with the use of adjuvant RA in patients scheduled for non-cardiothoracic surgery requiring ≥2 h general anesthesia with mechanical ventilation after adjusting for surgical and patient complexity variables.

## Methods

### Ethics approval and consent to participate

Institutional Review Board approval was obtained from the lead center of this sub-analysis (COMIRB# 14–0202) and was also obtained at each of the seven participating U.S. institutions. Either waiver of consent or an opt-out opportunity were approved as required by individual institutional review boards. All methods were carried out in accordance with relevant guidelines and regulations. This manuscript adheres to the Strengthening the Reporting of Observational Studies in Epidemiology (STROBE) guidelines for the reporting of observational studies [12].

### Design

This secondary analysis included all 1202 patients scheduled for non-cardiothoracic surgery requiring ≥2 h general anesthesia with mechanical ventilation who were participating in the original study [2]. The decision to use RA and which type of RA were not controlled in the original study. Therefore, for this sub-analysis patients were classified as receiving either RA + GA or GA alone and adjusted for surgical and patient complexity variables to test the hypothesis that the incidence of PPCs was not associated with the use of adjuvant RA.

### Subjects

Eligible patients included in the original study were prospectively identified from the regular operating room surgical schedule over a seven-month period [2]. Eligibility criteria included age ≥ 18 years, ASA class 3, any type of non-cardiothoracic surgery, planned GA with endotracheal intubation and mechanical ventilation, and an anticipated duration of the procedure of ≥2 hours. Exclusion criteria included long-term preoperative continuous ventilatory support, chronic oxygen therapy, tracheostomy, pregnancy, or a life expectancy ≤30 days.

### Interventions

Patients were not assigned any controlled interventions. Retrospectively, patients were classified as receiving either RA + GA or GA alone. Adjuvant RA included any techniques of neuraxial (e.g., epidural, spinal) or peripheral nerve blocks as administered by the patients’ respective clinicians. The selection of the specific RA technique was independent of the original study purposes. No additional details of individual techniques (e.g., single-shot vs. continuous infusion, types and doses of medications used) beyond their presence in conjunction with GA were collected in this seven-center observational study.

### Outcomes

A composite of predefined PPCs occurring within the first seven postoperative days were prospectively identified and included: pneumonia, bronchospasm, Acute Respiratory Distress Syndrome (ARDS), atelectasis, pneumothorax, pleural effusion, prolonged (> 1 day after end of surgery) supplemental oxygen by nasal cannula and/or facemask, postoperative noninvasive ventilation, and re-intubation with postoperative mechanical ventilation. This composite of PPCs was used in the original study [2] and it is based on previous studies and definitions [13–16]. Secondary outcomes included the individual type of PPC, any postoperative requirement for oxygen supplementation by nasal cannula or face mask, hospital length of stay, Intensive Care Unit (ICU) or intermediate care unit length of stay, and in-hospital mortality.

### Statistical analysis

Patient demographics, comorbidities, perioperative characteristics and clinical outcomes were summarized with mean (standard deviation) and n (%), and compared using hierarchical regression, clustering patients within sites. The statistical analysis for this sub-analysis follows the same methodological details as the one used in the original study after reorganization of the original dataset [2]. Bivariable and multivariable hierarchical logistic regression analyses were used to investigate the association of RA + GA (versus GA) with the occurrence of at least one PPC. Relevant covariates adjusted for in the model were those considered clinically relevant and unevenly distributed (*p* < 0.05) in the bivariable analysis, and if they had no significant statistical association with other relevant covariates. The linearity of each continuous predictor with the log odds of the outcome was checked graphically and, if not present, a variable log transformation was performed. Spearman and Pearson correlation coefficients were used to assess collinearity between predictors [2]. To examine the appropriateness of the final model, the intraclass correlation coefficient was calculated. The intraclass correlation coefficient represents the ratio of between-site variance to total variance and ranges from 0 to 1 (larger values represent stronger clustering effects) [2]. All analyses were performed using SAS version 9.4. All statistical tests were performed as two-sided tests with a level of significance of 0.05.

## Results

RA + GA was performed in 266 (22.1%) and GA alone in 936 (77.9%) patients. RA + GA patients were more likely to be older, have cancer, and were less likely to have neurological comorbidities compared to GA patients at baseline (Table 1). The presence of preoperative pulmonary comorbidities was similar in both groups (Table 1) and therefore none of them were included in the model. Preoperative oxygen saturation was significantly higher in RA + GA patients compared to GA patients, but the observed difference was not clinically meaningful (Table 2). Procedures in the RA + GA group were more likely to be non-emergent, abdominal or pelvic surgeries, involved increased blood loss and higher volume of administered colloids and crystalloids, and required more frequent use of non-depolarizing neuromuscular blockade (NMB), as well as NMB monitoring and NMB reversal (Table 2). RA + GA patients had a higher incidence of ≥1 PPC (42.1%) compared to GA patients (30.9%), site-adjusted *p* = 0.007 (Table 3). Specifically, atelectasis, pleural effusion, the requirement of postoperative oxygen supplementation by nasal cannula, and the prolonged (> 1d) requirement of postoperative oxygen supplementation (by nasal cannula or face mask) were more prevalent in RA + GA than GA patients (Table 3). Hospital length of stay was more prolonged in RA + GA patients compared to GA patients, but ICU/intermediate unit length of stay and in-hospital mortality were similar in both groups (Table 3).

**Table 1:** Demographics and comorbidities

Patient Characteristics	RA + GA (***n*** = 266)	GA (***n*** = 936)	adjusted ***P***-value for site clusters
Age, years	63.6 (11.5)	61.7 (14.3)	0.0177
Women	131 (49.2)	435 (46.5)	0.3940
Body Mass Index, kg/m^2^	29.1 (6.5)	30.2 (7.8)	0.0583
Cerebrovascular Disease	12 (4.5)	91 (9.7)	0.0606
Neurological Disease	36 (13.5)	229 (24.5)	0.0112
Hypertension	178 (66.9)	612 (65.4)	0.2890
Coronary Artery Disease	56 (21.1)	190 (20.3)	0.6606
Cardiac Valvular Disease	13 (4.9)	59 (6.3)	0.4424
Heart Failure	12 (4.5)	56 (6.0)	0.3745
Chronic Obstructive Pulmonary Disease	21 (7.9)	82 (8.8)	0.7469
Asthma	43 (16.2)	129 (13.8)	0.2554
Obstructive Sleep Apnea	42 (15.8)	192 (20.5)	0.2044
Current Smoking	32 (12.0)	129 (13.8)	0.3056
Former Smoking	120 (47.1)	358 (40.5)	0.1409
Cancer	146 (54.9)	353 (37.7)	0.0050
Gastro-Esophageal Reflux Disease	113 (42.5)	318 (34.0)	0.0501
Renal Disease	48 (18.0)	197 (21.0)	0.6283
Chronic Renal failure	24 (9.0)	114 (12.2)	0.3505
Liver Disease	37 (13.9)	109 (11.7)	0.1787
Diabetes Mellitus	74 (27.8)	227 (24.3)	0.1974
Thyroid Disease	38 (14.3)	161 (17.2)	0.4805
Alcohol Abuse	17 (6.4)	75 (8.0)	0.5263
(Data are presented as average (SD) or number (% of respective column), as appropriate)

**Table 2:** Perioperative characteristics

Variables	RA + GA (***n*** = 266)	GA (***n*** = 936)	adj ***P***-value for site clusters
Preoperative Peripheral Saturation of Oxyhemoglobin (SpO_2_), %	97.7 (2.0)	97.1 (2.2)	< 0.0001
Procedure Characteristics			
Emergency Surgery	4 (1.5)	57 (6.1)	0.0472
Abdominal/Pelvic Surgery	174 (65.4)	371 (39.6)	0.0003
Surgery Duration, hours	3.9 (2.1)	3.6 (2.0)	0.1034
Anesthesia Duration, hours	4.8 (2.2)	4.6 (2.2)	0.3486
Mechanical ventilation			
Ventilatory Modes			0.4295
Volume Controlled Ventilation	187 (70.3)	637 (68.1)	
Pressure Controlled Ventilation	47 (17.7)	131 (14.0)	
Assisted/Supported Ventilation	7 (2.6)	41 (4.4)	
Unspecified	25 (9.4)	126 (13.5)	
Median Exhaled Tidal Volume (VT), mL/kgPBW	8.0 (1.4)	8.0 (1.7)	0.1543
Median Inspired Fraction of Oxygen (FiO2), %	53.9 (12.1)	54.6 (14.3)	0.4305
Median End-Expiratory Pressure (PEEP), cmH_2_O	5 (1)	5 (2)	0.9420
Median Peak Inspiratory Pressure, cmH_2_O	20.7 (4.9)	21.4 (5.5)	0.0218
Fluid Balance			
Estimated Blood Loss, mL	456.8 (948)	310.5 (531)	0.0106
Urine Output, mL/kg/h	1.11 (1.26)	1.28 (1.61)	0.0750
Crystalloids (ml/kg/hr)	6.97 (3.89)	6.14 (3.59)	0.0027
Colloids (mL/kg/hr)	0.5 (1.2)	0.3 (0.7)	<.0001
Any Blood Product transfused	33 (12.4)	99 (10.6)	0.4243
Neuromuscular Blockade and Reversal Management			
Depolarizing neuromuscular blockade	97 (36.6)	343 (36.7)	0.9697
Non-Depolarizing neuromuscular blockade	245 (92.1)	793 (84.8)	0.0223
Neuromuscular blockade monitoring	194 (80.5)	555 (68.4)	0.0116
Neostigmine Dose, mcg/kg	37.1 (24.3)	29.9 (23.8)	< 0.0001
Hypnotics and Analgesics Administered (Any Dose)			
Propofol	98 (36.8)	413 (44.2)	0.0392
Fentanyl	257 (96.6)	857 (91.6)	0.0331
Hydromorphone	126 (47.4)	509 (54.4)	0.0222
Morphine	8 (3.0)	61 (6.5)	0.0942
Remifentanil	34 (12.8)	176 (18.8)	0.0064
Ketamine	24 (9.0)	135 (14.4)	0.1382

(Data are presented as average (SD) or number (% of respective column), as appropriate)

**Table 3:** Clinical outcomes

Variables	RA + GA (***n*** = 266)	GA (***n*** = 936)	adj ***P***-value for site clusters
At Least One Postoperative Pulmonary Complication	112 (42.1)	289 (30.9)	0.0066
Respiratory Failure	28 (10.6)	86 (9.2)	0.5478
ARDS	0 (0.0)	2 (0.2)	N/A
Pneumonia	8 (3.0)	14 (1.5)	0.1820
Pneumothorax	1 (0.4)	3 (0.3)	0.9187
Atelectasis	61 (22.9)	145 (15.5)	0.0238
Pleural Effusion	40 (15.0)	76 (8.1)	0.0139
Bronchospasm	3 (1.1)	10 (1.1)	0.7847
Postoperative Oxygen Supplementation > 1 day (Nasal Cannula or Face Mask)	69 (25.9)	173 (18.5)	0.0170
Postoperative Requirement of Non-Invasive Ventilation (N-INV)	12 (4.5)	34 (3.6)	0.3815
Postoperative Requirement of Intubation and Mechanical Ventilation	4 (1.5)	17 (1.8)	0.8217
Postoperative Oxygen Supplementation by Nasal Cannula, any duration	119 (45.1)	379 (40.9)	0.0213
Postoperative Oxygen Supplementation by Face Mask, any duration	4 (1.6)	42 (4.8)	0.0899
ICU/Intermediate Unit Length of Stay, days	0.7 (2.1)	0.8 (3.5)	0.6096
Hospital Length of Stay, days	8.0 (9.2)	5.6 (7.6)	0.0004
In-hospital Mortality	3 (1.1)	6 (0.6)	0.4425

*ARDS* Acute Respiratory Distress Syndrome, *ICU* Intensive Care Unit, *N-INV* Non-invasive ventilation

(Data are presented as average (SD) or number (% of respective column), as appropriate)

The association of RA + GA with ≥1 PPC was not significant (adjusted OR 1.37; 95% CI, 0.83–2.25; *p* = 0.165) after adjusting for age, pre-existing neurological disease, preoperative SpO_2_, procedure characteristics (emergency surgery, abdominal/pelvic surgery), intraoperative medications, intraoperative parameters related to fluid balance (e.g. blood loss), and neuromuscular blockade. Of note, age, preoperative SpO_2_, emergency surgery, abdominal/pelvic surgery, blood loss and the administration of any colloid were still significantly associated with ≥1 PPC following adjustment for covariates (Table 4). The estimated intraclass correlation coefficient (ICC) of the hierarchical model indicates that the ratio of between-site variance to total variance is 3.8%, which indicates modest variability between hospitals. The model was considered with and without NMB monitoring due to its large percentage of missing (*n* = 150 (12.5%)) and moderate correlation with non-depolarizing NMB (rho = 0.47). The magnitude and significance of the odds ratio of the primary relationship of interest was not impacted by NMB monitoring as a covariate.

**Table 4:** Hierarchical multivariable logistic regression models to investigate the association of adjuvant regional anesthesia (RA + GA) versus general anesthesia only (GA) with the occurrence of at least one PPC

Variable	Crude Models (adjusted for site)			Adjusted Model (adjusted for site and other covariates)			
	OR	Lower 95%CI	Upper 95%CI	OR	Lower 95%CI	Upper 95%CI	***P***-value
RA + GA	1.827	1.270	2.629	1.368	0.834	2.246	0.1646
Neurological disease	0.881	0.603	1.287	1.140	0.683	1.902	0.5408
Emergency	2.160	1.069	4.366	3.040	1.091	8.469	0.0394
Abdominal/Pelvic Surgery	2.515	1.837	3.444	2.956	1.854	4.712	0.0019
Non-Depolarizing neuromuscular blockade	1.608	0.997	2.594	1.167	0.526	2.588	0.6398
Neuromuscular blockade monitoring	1.293	0.869	1.924	0.794	0.462	1.362	0.3213
Propofol	0.829	0.548	1.255	1.055	0.581	1.915	0.8168
Colloid	3.291	2.194	4.937	1.999	1.137	3.513	0.0252
Hydromorphone	0.966	0.656	1.423	0.957	0.554	1.651	0.8332
Fentanyl	1.194	0.648	2.198	1.392	0.609	3.183	0.3509
Remifentanil	0.764	0.489	1.193	1.145	0.652	2.010	0.5638
Age, years	1.027	1.018	1.037	1.031	1.018	1.044	< 0.0001
Log of Estimated Blood Loss, mL	1.322	1.217	1.435	1.180	1.068	1.303	0.0012
Preoperative Peripheral Saturation of Oxyhemoglobin (SpO_2_), %	0.893	0.840	0.949	0.836	0.774	0.903	< 0.0001
Crystalloid	1.038	1.005	1.073	1.017	0.975	1.060	0.4405
Neostigmine Dose, mcg/kg	1.009	1.003	1.014	1.000	0.992	1.007	0.9524

Regression modeling used site as a random effect, which allows for clustering of patients within hospitals. Crude odds ratios adjusted for site were examined, and important covariates (identified as those with clinical relevance and *p*-values less than 0.05 in the bivariable analyses) were added to include in the final adjusted model

## Discussion

Adyuvant RA is often employed to mitigate the risk of postoperative pulmonary complications (PPCs) after general anesthesia in susceptible patients undergoing high-risk surgeries. Surprisingly, our multicenter observational study [2] found a higher occurrence of at least one PPC in non-cardiothoracic surgical patients with severe systemic disease (ASA physical status 3) receiving adjuvant regional anesthesia (RA + GA) compared to patients receiving GA alone. However, the current sub-analysis showed that adjuvant RA was not associated with the occurrence of PPCs after adjusting for covariates reflecting surgical complexity and patient-specific risk factors for PPCs.

The prevalence of PPCs observed was 30.9% in patients with GA alone and 42.1% in RA + GA patients. Given their association with worse clinical outcomes and higher costs [2], identifying risk factors to design targeted interventions to reduce PPCs is critical.

The contribution of general anesthesia to the development of PPCs is multifactorial. After induction of general anesthesia, functional residual capacity decreases, diaphragmatic excursion is altered and ventilation/perfusion mismatch increases, promoting shunting, atelectasis and hypoxemia [5, 17–19]. General anesthesia also inhibits mucociliary clearance and surfactant release, reduces the number and function of alveolar macrophages, and increases alveolar-capillary permeability and the sensitivity of the pulmonary vasculature to neurohumoral mediators [5]. All these changes contribute to the development of atelectasis, different degrees of respiratory insufficiency, and pneumonia. Yet, general anesthesia is mandatory for some types of surgical procedures, such as major abdominal surgery and most neurosurgical procedures. Identifying modifiable factors that could reduce the incidence of PPCs when general anesthesia is unavoidable would likely help improve clinical outcomes.

The RA + GA group included more abdominal/pelvic surgeries and fewer emergency procedures than the GA group. Adjuvant RA is often combined with GA in thoracic, abdominal, pelvic and peripheral surgeries to reduce the requirement of intravenous opioid-based analgesics and other medications (e.g., hypnotics) that can contribute to residual respiratory depression after surgery. The overall goal of reducing intravenous opioids and their side effects, including respiratory depression, is a consistent theme of all Enhanced Recovery After Surgery (ERAS) protocols being developed for various surgeries [20, 21]. The specific RA technique to achieve this goal (e.g., epidural, intrathecal, or peripheral nerve blocks) depends on the surgical procedure, patient eligibility, and institutional practices. The original study [2] included a variety of procedures and patient characteristics, and the selection of the RA technique was not pre-specified by the study protocol or reported in detail. Therefore, ascertaining any unique effects of individual RA approaches is beyond the limits of this sub-analysis.

Several studies have reported improved respiratory function postoperatively by regional analgesia compared to intravenous (opioid-based) analgesia [6, 7, 22, 23]. For example, patients receiving epidural administration of local anesthetics, a common RA used for abdominal/pelvic procedures, achieved higher postoperative vital capacity (VC), forced expiratory volume at 1 minute (FEV1) and arterial oxygenation compared to surgical patients recovering from general anesthesia without an epidural block [6, 22, 23]. A large meta-analysis including 141 randomized trials and 9559 patients found that neuraxial blockade reduced respiratory depression by 59% and pneumonia by 39% (both *P* < 0.001) [4]. A subsequent meta-analysis of 58 trials and 5904 patients suggested that epidural analgesia protected against pneumonia following abdominal or thoracic surgery, but that this beneficial effect had lessened over time [23]. More recently, a Cochrane systematic review concluded that the addition of neuraxial blockade to general anesthesia reduced the risk of postoperative pneumonia and 30-day mortality [24].

Despite these positive results, the impact of using RA techniques to reduce PPCs or improve overall clinical outcomes after general anesthesia, compared to patients receiving intravenous analgesia, has not been consistently demonstrated [5, 25–28]. It is possible that diaphragmatic function can be impaired from certain RA techniques, and this may have influenced the conflicting results in terms of PPCs. For instance, thoracic epidural anesthesia itself can reduce VC and FEV_1_ by 15 to 20% of baseline [6]. Similarly, abdominal surgery alone can decrease VC by 60% or more and it may not be completely returned to baseline levels for up to 14 days [6].

The observation in our original study that RA + GA was associated with the occurrence of at least one PPC [2] was nonetheless unexpected and counterintuitive. Indeed, this finding sparked off this sub-analysis. Our current study revealed that RA + GA was no longer associated with the occurrence of PPCs after further adjusting for variables reflecting patient risk, surgical complexity and other confounding factors. Risk factors associated with the occurrence of PPCs in this sub-analysis included age, emergency condition, abdominal/pelvic surgery, any colloid administration (only albumin was used), estimated blood loss and preoperative oxyhemoglobin saturation. These risk factors have also been observed in various prior studies [2, 13, 29]. The intraoperative administration of albumin as intravenous colloid fluid was the only modifiable risk factor associated with the presence of at least one PPC in this sub-analysis. However, it may be that the administration of albumin is primarily a marker for procedure complexity. Knowledge of these risk factors may allow improved risk stratification and design of prospective studies aiming to reduce the incidence of PPCs.

Our study presents several important limitations, primarily related to the observational nature of the original study and the chosen methodologic approach of performing a secondary analysis of an existing data set. Remaining biases related to the selection of patients or the care they received remains possible. Yet, we attempted to address this issue by building an appropriate regression model and adjusting for the most relevant risk factors. Second, only the use of any adjuvant RA was recorded but not the individual technique (e.g. epidural, spinal, or peripheral nerve block). Therefore, unique effects of individual adjuvant RA techniques could not be ascertained. Third, there is no uniform composite and consistent definition for PPCs, which may limit comparing our findings to other studies. However, our definition of PPCs was based on landmark studies by others [13, 14, 16], and thereby reflects current approaches in the field.

## Conclusions

Our findings suggest that, after adjusting for patient and surgical complexity risk factors, adjuvant RA was not significantly associated with increased PPCs in the original study including ASA physical status 3 adult patients undergoing non-cardiothoracic surgery under GA. We identified a higher risk for PPCs in patients with low preoperative oxygenation (SpO_2_), patients undergoing emergency surgery or abdominal/pelvic surgery, and those with high blood loss. Future studies should confirm this observation stratified by the specific type of regional anesthetic techniques used. Mitigating PPCs in high-risk populations remains a priority in perioperative medicine research.

## Data Availability

Data used for this secondary analysis was published in Fernandez-Bustamante et al., JAMA Surgery 2017 (PMCID #5334462). Data are available from the authors upon reasonable request. Please refer any data requests to Dr. Fernandez-Bustamante at Ana.Fernandez-Bustamante@cuanschutz.edu.

## Abbreviations

ARDS: Acute Respiratory Distress Syndrome
ASA: American Society of Anesthesiologists physical status
ERAS: Enhanced Recovery After Surgery
FEV1: Forced Expiratory Volume at 1 minute
ICC: Intraclass Correlation Coeficient
ICU: Intensive Care Unit
GA: General Anesthesia
NMB: Neuromuscular Blockade
OR: Odds Ratio
PPC: Postoperative Pulmonary Complication
PRN: Perioperative Research Network
RA: Regional Analgesia
SpO_2_: Peripheral saturation of oxyhemoglobin
VC: Vital Capacity

## References

[1] Shander A, Fleisher LA, Barie PS, Bigatello LM, Sladen RN, Watson CB (2011). Clinical and economic burden of postoperative pulmonary complications: patient safety summit on definition, risk-reducing interventions, and preventive strategies. Crit Care Med.

[2] Fernandez-Bustamante A, Frendl G, Sprung J, Kor DJ, Subramaniam B, Martinez Ruiz R, Lee JW, Henderson WG, Moss A, Mehdiratta N (2017). Postoperative pulmonary complications, early mortality, and hospital stay following noncardiothoracic surgery: a multicenter study by the perioperative research network investigators. JAMA Surg.

[3] Haines KL, Agarwal S (2017). Postoperative pulmonary complications-a multifactorial outcome. JAMA Surg.

[4] Rodgers A, Walker N, Schug S, McKee A, Kehlet H, van Zundert A, Sage D, Futter M, Saville G, Clark T (2000). Reduction of postoperative mortality and morbidity with epidural or spinal anaesthesia: results from overview of randomised trials. BMJ.

[5] Rock P, Rich PB (2003). Postoperative pulmonary complications. Curr Opin Anaesthesiol.

[6] Groeben H (2006). Epidural anesthesia and pulmonary function. J Anesth.

[7] Rigg JR, Jamrozik K, Myles PS, Silbert BS, Peyton PJ, Parsons RW, Collins KS, Group MATS (2002). Epidural anaesthesia and analgesia and outcome of major surgery: a randomised trial. Lancet.

[8] Park WY, Thompson JS, Lee KK (2001). Effect of epidural anesthesia and analgesia on perioperative outcome: a randomized, controlled veterans affairs cooperative study. Ann Surg.

[9] Richman JM, Liu SS, Courpas G, Wong R, Rowlingson AJ, McGready J, Cohen SR, Wu CL (2006). Does continuous peripheral nerve block provide superior pain control to opioids? A meta-analysis. Anesth Analg.

[10] Fredrickson MJ, Krishnan S, Chen CY (2010). Postoperative analgesia for shoulder surgery: a critical appraisal and review of current techniques. Anaesthesia.

[11] Kettner SC, Willschke H, Marhofer P (2011). Does regional anaesthesia really improve outcome?. Br J Anaesth.

[12] von Elm E, Altman DG, Egger M, Pocock SJ, Gotzsche PC, Vandenbroucke JP, Initiative S (2014). The strengthening the reporting of observational studies in epidemiology (STROBE) statement: guidelines for reporting observational studies. Int J Surg.

[13] Canet J, Gallart L, Gomar C, Paluzie G, Valles J, Castillo J, Sabate S, Mazo V, Briones Z, Sanchis J (2010). Prediction of postoperative pulmonary complications in a population-based surgical cohort. Anesthesiology.

[14] Cassidy MR, Rosenkranz P, McCabe K, Rosen JE, McAneny D (2013). I COUGH: reducing postoperative pulmonary complications with a multidisciplinary patient care program. JAMA Surg.

[15] Futier E, Constantin JM, Paugam-Burtz C, Pascal J, Eurin M, Neuschwander A, Marret E, Beaussier M, Gutton C, Lefrant JY (2013). A trial of intraoperative low-tidal-volume ventilation in abdominal surgery. N Engl J Med.

[16] Force ADT, Ranieri VM, Rubenfeld GD, Thompson BT, Ferguson ND, Caldwell E, Fan E, Camporota L, Slutsky AS (2012). Acute respiratory distress syndrome: the Berlin definition. JAMA.

[17] Bigatello L, Pesenti A (2019). Respiratory physiology for the anesthesiologist. Anesthesiology.

[18] Rehder K, Sessler AD, Marsh HM (1975). General anesthesia and the lung. Am Rev Respir Dis.

[19] Bendixen HH, Hedley-Whyte J, Laver MB (1963). Impaired oxygenation in surgical patients during general anesthesia with controlled ventilation. A concept of atelectasis. N Engl J Med.

[20] Wu CL, King AB, Geiger TM, Grant MC, Grocott MPW, Gupta R, Hah JM, Miller TE, Shaw AD, Gan TJ (2019). American Society for Enhanced Recovery and Perioperative Quality Initiative Joint Consensus Statement on perioperative opioid minimization in opioid-naive patients. Anesth Analg.

[21] Scott MJ, McEvoy MD, Gordon DB, Grant SA, Thacker JKM, Wu CL, Gan TJ, Mythen MG, Shaw AD, Miller TE (2017). American Society for Enhanced Recovery (ASER) and perioperative quality Initiative (POQI) joint consensus statement on optimal analgesia within an enhanced recovery pathway for colorectal surgery: part 2-from PACU to the transition home. Perioper Med (Lond).

[22] Jayr C, Thomas H, Rey A, Farhat F, Lasser P, Bourgain JL (1993). Postoperative pulmonary complications. Epidural analgesia using bupivacaine and opioids versus parenteral opioids. Anesthesiology.

[23] Popping DM, Elia N, Marret E, Remy C, Tramer MR (2008). Protective effects of epidural analgesia on pulmonary complications after abdominal and thoracic surgery: a meta-analysis. Arch Surg.

[24] Guay J, Choi P, Suresh S, Albert N, Kopp S, Pace NL (2014). Neuraxial blockade for the prevention of postoperative mortality and major morbidity: an overview of Cochrane systematic reviews. Cochrane Database Syst Rev.

[25] Wijeysundera DN, Beattie WS, Austin PC, Hux JE, Laupacis A (2008). Epidural anaesthesia and survival after intermediate-to-high risk non-cardiac surgery: a population-based cohort study. Lancet.

[26] Li J, Pourrahmat MM, Vasilyeva E, Kim PT, Osborn J, Wiseman SM (2019). Efficacy and safety of patient-controlled analgesia compared with epidural analgesia after open hepatic resection: a systematic review and Meta-analysis. Ann Surg.

[27] Allen S, DeRoche A, Adams L, Slocum KV, Clark CJ, Fino NF, Shen P (2017). Effect of epidural compared to patient-controlled intravenous analgesia on outcomes for patients undergoing liver resection for neoplastic disease. J Surg Oncol.

[28] Leslie K, McIlroy D, Kasza J, Forbes A, Kurz A, Khan J, Meyhoff CS, Allard R, Landoni G, Jara X (2016). Neuraxial block and postoperative epidural analgesia: effects on outcomes in the POISE-2 trialdagger. Br J Anaesth.

[29] Brooks-Brunn JA (1997). Predictors of postoperative pulmonary complications following abdominal surgery. Chest.

